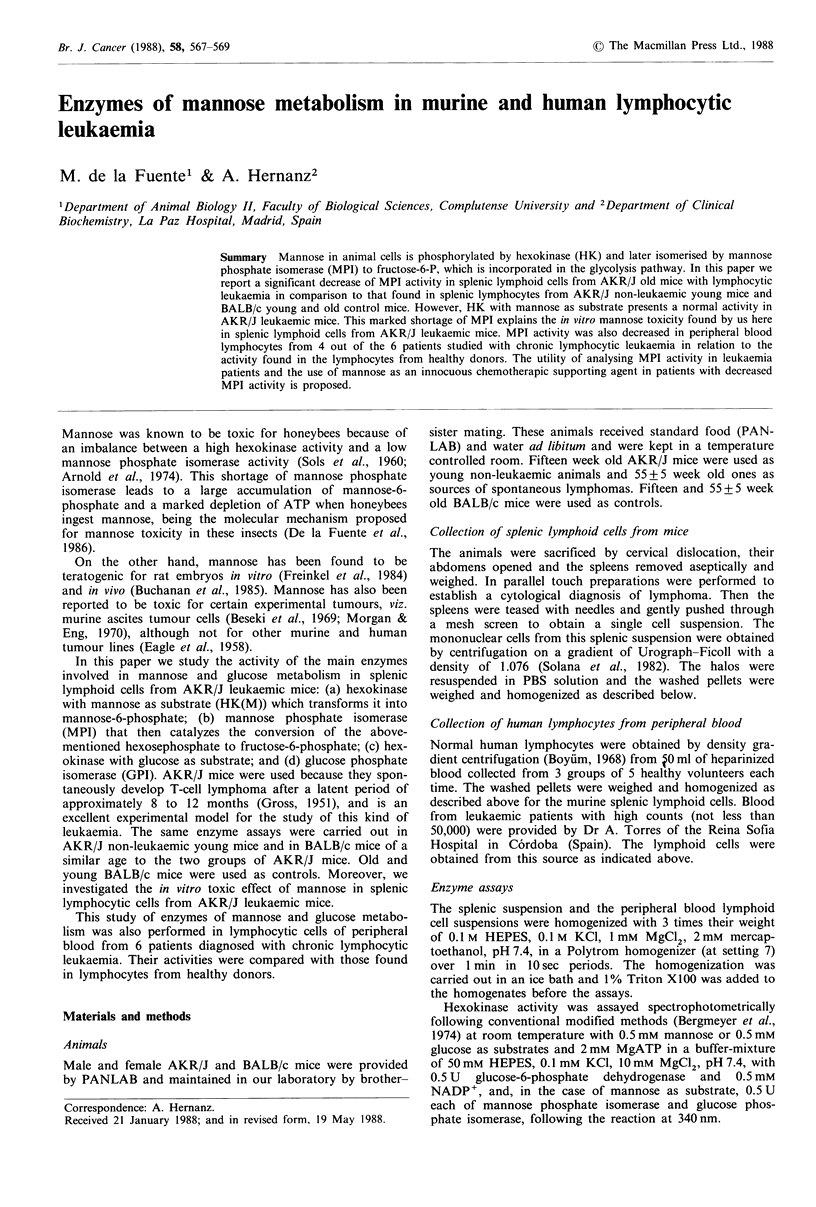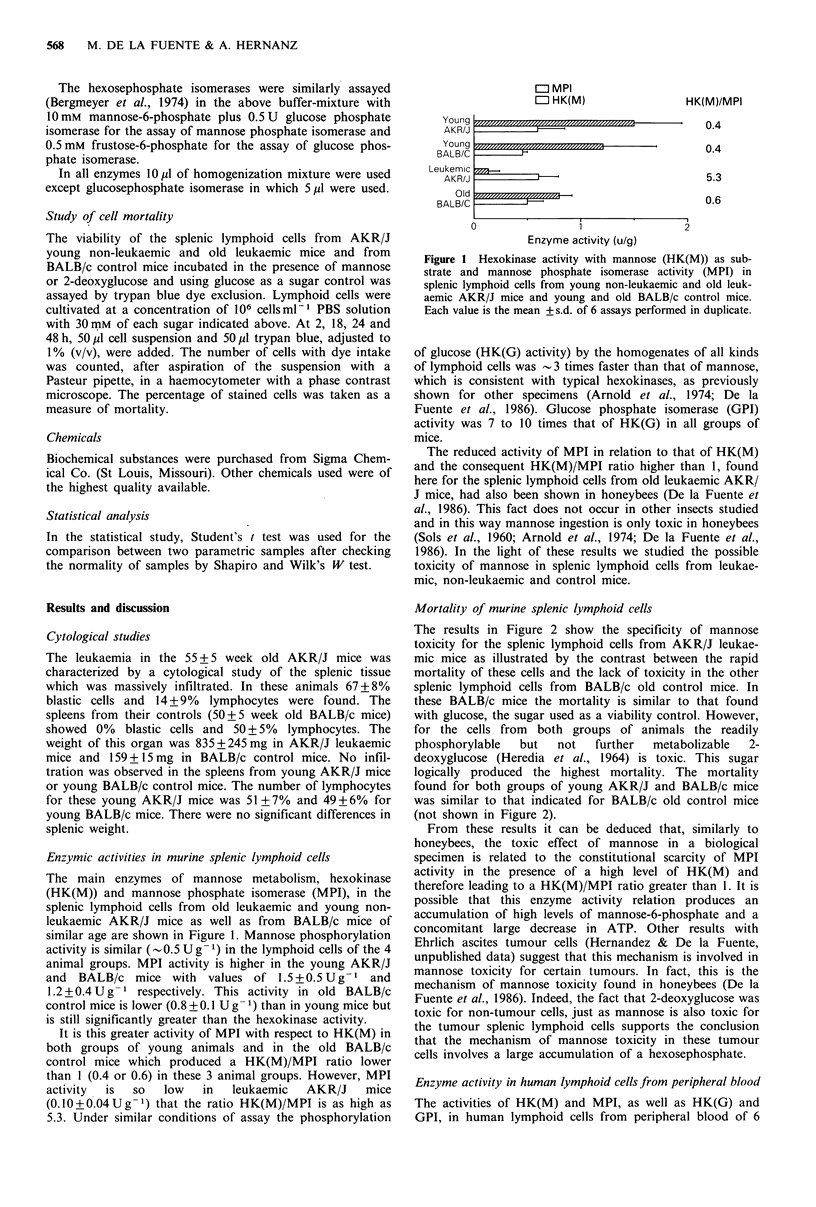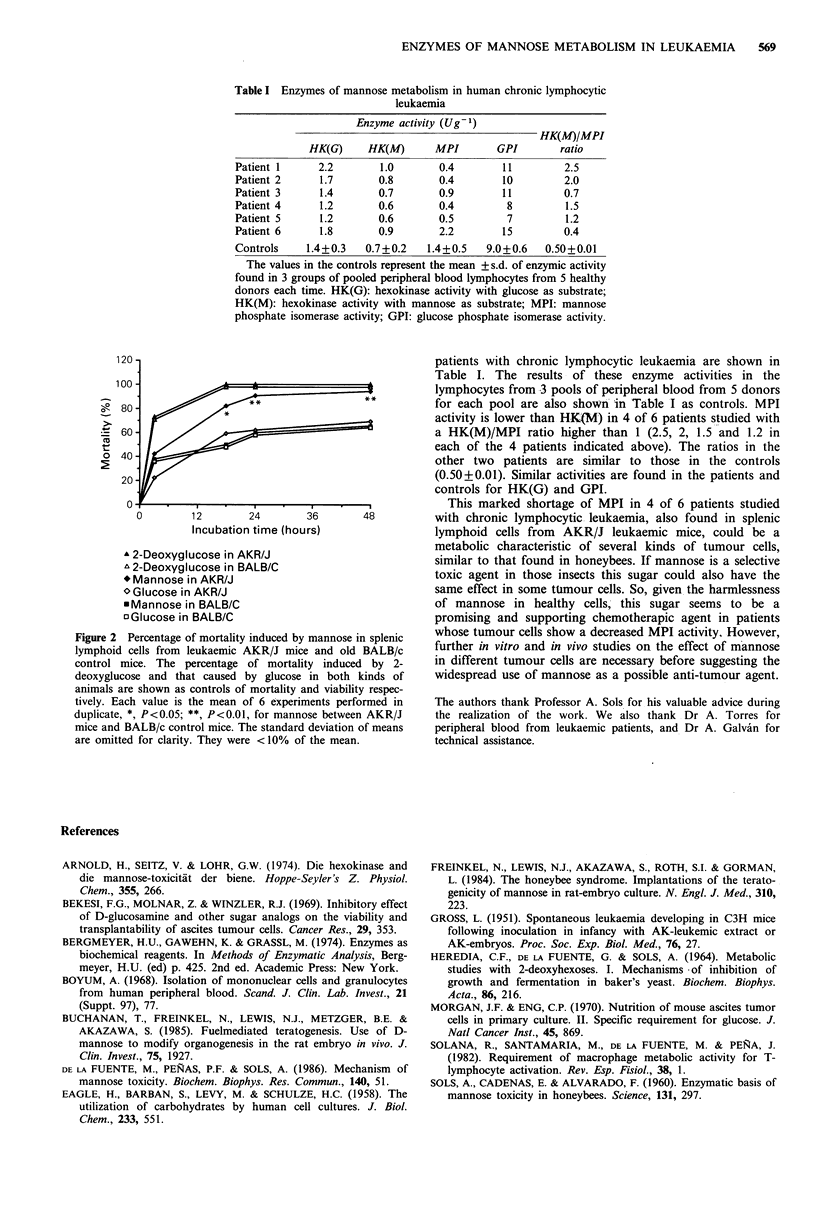# Enzymes of mannose metabolism in murine and human lymphocytic leukaemia.

**DOI:** 10.1038/bjc.1988.260

**Published:** 1988-11

**Authors:** M. de la Fuente, A. Hernanz

**Affiliations:** Department of Animal Biology II, Faculty of Biological Sciences, Complutense University, Madrid, Spain.

## Abstract

Mannose in animal cells is phosphorylated by hexokinase (HK) and later isomerised by mannose phosphate isomerase (MPI) to fructose-6-P, which is incorporated in the glycolysis pathway. In this paper we report a significant decrease of MPI activity in splenic lymphoid cells from AKR/J old mice with lymphocytic leukaemia in comparison to that found in splenic lymphocytes from AKR/J non-leukaemic young mice and BALB/c young and old control mice. However, HK with mannose as substrate presents a normal activity in AKR/J leukaemic mice. This marked shortage of MPI explains the in vitro mannose toxicity found by us here in splenic lymphoid cells from AKR/J leukaemic mice. MPI activity was also decreased in peripheral blood lymphocytes from 4 out of the 6 patients studied with chronic lymphocytic leukaemia in relation to the activity found in the lymphocytes from healthy donors. The utility of analysing MPI activity in leukaemia patients and the use of mannose as an innocuous chemotherapic supporting agent in patients with decreased MPI activity is proposed.


*Br. J. Cancer* (1988), **58**, 567–569 © The Macmillan Press Ltd., 1988

### Enzymes of mannose metabolism in murine and human lymphocytic leukaemia

M. de la Fuente[1] & A. Hernanz[2]

[1]*Department of Animal Biology II, Faculty of Biological Sciences, Complutense University and* [2]*Department of Clinical Biochemistry, La Paz Hospital, Madrid, Spain*

**Summary** Mannose in animal cells is phosphorylated by hexokinase (HK) and later isomerised by mannose phosphate isomerase (MPI) to fructose-6-P, which is incorporated in the glycolysis pathway. In this paper we report a significant decrease of MPI activity in splenic lymphoid cells from AKR/J old mice with lymphocytic leukaemia in comparison to that found in splenic lymphocytes from AKR/J non-leukaemic young mice and BALB/c young and old control mice. However, HK with mannose as substrate presents a normal activity in AKR/J leukaemic mice. This marked shortage of MPI explains the *in vitro* mannose toxicity found by us here in splenic lymphoid cells from AKR/J leukaemic mice. MPI activity was also decreased in peripheral blood lymphocytes from 4 out of the 6 patients studied with chronic lymphocytic leukaemia in relation to the activity found in the lymphocytes from healthy donors. The utility of analysing MPI activity in leukaemia patients and the use of mannose as an innocuous chemotherapic supporting agent in patients with decreased MPI activity is proposed.

Mannose was known to be toxic for honeybees because of an imbalance between a high hexokinase activity and a low mannose phosphate isomerase activity (Sols *et al.*, 1960; Arnold *et al.*, 1974). This shortage of mannose phosphate isomerase leads to a large accumulation of mannose-6-phosphate and a marked depletion of ATP when honeybees ingest mannose, being the molecular mechanism proposed for mannose toxicity in these insects (De la Fuente *et al.*, 1986).

On the other hand, mannose has been found to be teratogenic for rat embryos *in vitro* (Freinkel *et al.*, 1984) and *in vivo* (Buchanan *et al.*, 1985). Mannose has also been reported to be toxic for certain experimental tumours, *viz.* murine ascites tumour cells (Beseki *et al.*, 1969; Morgan & Eng, 1970), although not for other murine and human tumour lines (Eagle *et al.*, 1958).

In this paper we study the activity of the main enzymes involved in mannose and glucose metabolism in splenic lymphoid cells from AKR/J leukaemic mice: (a) hexokinase with mannose as substrate (HK(M)) which transforms it into mannose-6-phosphate; (b) mannose phosphate isomerase (MPI) that then catalyzes the conversion of the above-mentioned hexosephosphate to fructose-6-phosphate; (c) hexokinase with glucose as substrate; and (d) glucose phosphate isomerase (GPI). AKR/J mice were used because they spontaneously develop T-cell lymphoma after a latent period of approximately 8 to 12 months (Gross, 1951), and is an excellent experimental model for the study of this kind of leukaemia. The same enzyme assays were carried out in AKR/J non-leukaemic young mice and in BALB/c mice of a similar age to the two groups of AKR/J mice. Old and young BALB/c mice were used as controls. Moreover, we investigated the *in vitro* toxic effect of mannose in splenic lymphocytic cells from AKR/J leukaemic mice.

This study of enzymes of mannose and glucose metabolism was also performed in lymphocytic cells of peripheral blood from 6 patients diagnosed with chronic lymphocytic leukaemia. Their activities were compared with those found in lymphocytes from healthy donors.

### Materials and methods

### *Animals*

Male and female AKR/J and BALB/c mice were provided by PANLAB and maintained in our laboratory by brother-sister mating. These animals received standard food (PANLAB) and water *ad libitum* and were kept in a temperature controlled room. Fifteen week old AKR/J mice were used as young non-leukaemic animals and $55 \pm 5$ week old ones as sources of spontaneous lymphomas. Fifteen and $55 \pm 5$ week old BALB/c mice were used as controls.

### *Collection of splenic lymphoid cells from mice*

The animals were sacrificed by cervical dislocation, their abdomens opened and the spleens removed aseptically and weighed. In parallel touch preparations were performed to establish a cytological diagnosis of lymphoma. Then the spleens were teased with needles and gently pushed through a mesh screen to obtain a single cell suspension. The mononuclear cells from this splenic suspension were obtained by centrifugation on a gradient of Urograph–Ficoll with a density of 1.076 (Solana *et al.*, 1982). The halos were resuspended in PBS solution and the washed pellets were weighed and homogenized as described below.

### *Collection of human lymphocytes from peripheral blood*

Normal human lymphocytes were obtained by density gradient centrifugation (Boyüm, 1968) from 50 ml of heparinized blood collected from 3 groups of 5 healthy volunteers each time. The washed pellets were weighed and homogenized as described above for the murine splenic lymphoid cells. Blood from leukaemic patients with high counts (not less than 50,000) were provided by Dr A. Torres of the Reina Sofia Hospital in Córdoba (Spain). The lymphoid cells were obtained from this source as indicated above.

### *Enzyme assays*

The splenic suspension and the peripheral blood lymphoid cell suspensions were homogenized with 3 times their weight of 0.1 M HEPES, 0.1 M KCl, 1 mM $MgCl_2$, 2 mM mercaptoethanol, pH 7.4, in a Polytrom homogenizer (at setting 7) over 1 min in 10 sec periods. The homogenization was carried out in an ice bath and 1% Triton X100 was added to the homogenates before the assays.

Hexokinase activity was assayed spectrophotometrically following conventional modified methods (Bergmeyer *et al.*, 1974) at room temperature with 0.5 mM mannose or 0.5 mM glucose as substrates and 2 mM MgATP in a buffer-mixture of 50 mM HEPES, 0.1 mM KCl, 10 mM $MgCl_2$, pH 7.4, with 0.5 U glucose-6-phosphate dehydrogenase and 0.5 mM $NADP^+$, and, in the case of mannose as substrate, 0.5 U each of mannose phosphate isomerase and glucose phosphate isomerase, following the reaction at 340 nm.

Correspondence: A. Hernanz.
Received 21 January 1988; and in revised form, 19 May 1988.

The hexosephosphate isomerases were similarly assayed (Bergmeyer *et al.*, 1974) in the above buffer-mixture with 10 mM mannose-6-phosphate plus 0.5 U glucose phosphate isomerase for the assay of mannose phosphate isomerase and 0.5 mM frustose-6-phosphate for the assay of glucose phosphate isomerase.

In all enzymes 10 $\mu$l of homogenization mixture were used except glucosephosphate isomerase in which 5 $\mu$l were used.

*Study of cell mortality*

The viability of the splenic lymphoid cells from AKR/J young non-leukaemic and old leukaemic mice and from BALB/c control mice incubated in the presence of mannose or 2-deoxyglucose and using glucose as a sugar control was assayed by trypan blue dye exclusion. Lymphoid cells were cultivated at a concentration of $10^6$ cells $ml^{-1}$ PBS solution with 30 mM of each sugar indicated above. At 2, 18, 24 and 48 h, 50 $\mu$l cell suspension and 50 $\mu$l trypan blue, adjusted to 1% (v/v), were added. The number of cells with dye intake was counted, after aspiration of the suspension with a Pasteur pipette, in a haemocytometer with a phase contrast microscope. The percentage of stained cells was taken as a measure of mortality.

*Chemicals*

Biochemical substances were purchased from Sigma Chemical Co. (St Louis, Missouri). Other chemicals used were of the highest quality available.

*Statistical analysis*

In the statistical study, Student's *t* test was used for the comparison between two parametric samples after checking the normality of samples by Shapiro and Wilk's *W* test.

**Results and discussion**

*Cytological studies*

The leukaemia in the 55±5 week old AKR/J mice was characterized by a cytological study of the splenic tissue which was massively infiltrated. In these animals 67±8% blastic cells and 14±9% lymphocytes were found. The spleens from their controls (50±5 week old BALB/c mice) showed 0% blastic cells and 50±5% lymphocytes. The weight of this organ was 835±245 mg in AKR/J leukaemic mice and 159±15 mg in BALB/c control mice. No infiltration was observed in the spleens from young AKR/J mice or young BALB/c control mice. The number of lymphocytes for these young AKR/J mice was 51±7% and 49±6% for young BALB/c mice. There were no significant differences in splenic weight.

*Enzymic activities in murine splenic lymphoid cells*

The main enzymes of mannose metabolism, hexokinase (HK(M)) and mannose phosphate isomerase (MPI), in the splenic lymphoid cells from old leukaemic and young non-leukaemic AKR/J mice as well as from BALB/c mice of similar age are shown in Figure 1. Mannose phosphorylation activity is similar (~0.5 U $g^{-1}$) in the lymphoid cells of the 4 animal groups. MPI activity is higher in the young AKR/J and BALB/c mice with values of 1.5±0.5 U $g^{-1}$ and 1.2±0.4 U $g^{-1}$ respectively. This activity in old BALB/c control mice is lower (0.8±0.1 U $g^{-1}$) than in young mice but is still significantly greater than the hexokinase activity.

It is this greater activity of MPI with respect to HK(M) in both groups of young animals and in the old BALB/c control mice which produced a HK(M)/MPI ratio lower than 1 (0.4 or 0.6) in these 3 animal groups. However, MPI activity is so low in leukaemic AKR/J mice (0.10±0.04 U $g^{-1}$) that the ratio HK(M)/MPI is as high as 5.3. Under similar conditions of assay the phosphorylation

**Figure 1** Hexokinase activity with mannose (HK(M)) as substrate and mannose phosphate isomerase activity (MPI) in splenic lymphoid cells from young non-leukaemic and old leukaemic AKR/J mice and young and old BALB/c control mice. Each value is the mean ±s.d. of 6 assays performed in duplicate.

of glucose (HK(G) activity) by the homogenates of all kinds of lymphoid cells was ~3 times faster than that of mannose, which is consistent with typical hexokinases, as previously shown for other specimens (Arnold *et al.*, 1974; De la Fuente *et al.*, 1986). Glucose phosphate isomerase (GPI) activity was 7 to 10 times that of HK(G) in all groups of mice.

The reduced activity of MPI in relation to that of HK(M) and the consequent HK(M)/MPI ratio higher than 1, found here for the splenic lymphoid cells from old leukaemic AKR/J mice, had also been shown in honeybees (De la Fuente *et al.*, 1986). This fact does not occur in other insects studied and in this way mannose ingestion is only toxic in honeybees (Sols *et al.*, 1960; Arnold *et al.*, 1974; De la Fuente *et al.*, 1986). In the light of these results we studied the possible toxicity of mannose in splenic lymphoid cells from leukaemic, non-leukaemic and control mice.

*Mortality of murine splenic lymphoid cells*

The results in Figure 2 show the specificity of mannose toxicity for the splenic lymphoid cells from AKR/J leukaemic mice as illustrated by the contrast between the rapid mortality of these cells and the lack of toxicity in the other splenic lymphoid cells from BALB/c old control mice. In these BALB/c mice the mortality is similar to that found with glucose, the sugar used as a viability control. However, for the cells from both groups of animals the readily phosphorylable but not further metabolizable 2-deoxyglucose (Heredia *et al.*, 1964) is toxic. This sugar logically produced the highest mortality. The mortality found for both groups of young AKR/J and BALB/c mice was similar to that indicated for BALB/c old control mice (not shown in Figure 2).

From these results it can be deduced that, similarly to honeybees, the toxic effect of mannose in a biological specimen is related to the constitutional scarcity of MPI activity in the presence of a high level of HK(M) and therefore leading to a HK(M)/MPI ratio greater than 1. It is possible that this enzyme activity relation produces an accumulation of high levels of mannose-6-phosphate and a concomitant large decrease in ATP. Other results with Ehrlich ascites tumour cells (Hernandez & De la Fuente, unpublished data) suggest that this mechanism is involved in mannose toxicity for certain tumours. In fact, this is the mechanism of mannose toxicity found in honeybees (De la Fuente *et al.*, 1986). Indeed, the fact that 2-deoxyglucose was toxic for non-tumour cells, just as mannose is also toxic for the tumour splenic lymphoid cells supports the conclusion that the mechanism of mannose toxicity in these tumour cells involves a large accumulation of a hexosephosphate.

*Enzyme activity in human lymphoid cells from peripheral blood*

The activities of HK(M) and MPI, as well as HK(G) and GPI, in human lymphoid cells from peripheral blood of 6

**Table I** Enzymes of mannose metabolism in human chronic lymphocytic leukaemia

| | *Enzyme activity* ($Ug^{-1}$) | | | | |
|---|---|---|---|---|---|
| | *HK(G)* | *HK(M)* | *MPI* | *GPI* | *HK(M)/MPI ratio* |
| Patient 1 | 2.2 | 1.0 | 0.4 | 11 | 2.5 |
| Patient 2 | 1.7 | 0.8 | 0.4 | 10 | 2.0 |
| Patient 3 | 1.4 | 0.7 | 0.9 | 11 | 0.7 |
| Patient 4 | 1.2 | 0.6 | 0.4 | 8 | 1.5 |
| Patient 5 | 1.2 | 0.6 | 0.5 | 7 | 1.2 |
| Patient 6 | 1.8 | 0.9 | 2.2 | 15 | 0.4 |
| Controls | 1.4±0.3 | 0.7±0.2 | 1.4±0.5 | 9.0±0.6 | 0.50±0.01 |

The values in the controls represent the mean ±s.d. of enzymic activity found in 3 groups of pooled peripheral blood lymphocytes from 5 healthy donors each time. HK(G): hexokinase activity with glucose as substrate; HK(M): hexokinase activity with mannose as substrate; MPI: mannose phosphate isomerase activity; GPI: glucose phosphate isomerase activity.

**Figure 2** Percentage of mortality induced by mannose in splenic lymphoid cells from leukaemic AKR/J mice and old BALB/c control mice. The percentage of mortality induced by 2-deoxyglucose and that caused by glucose in both kinds of animals are shown as controls of mortality and viability respectively. Each value is the mean of 6 experiments performed in duplicate, *, $P<0.05$; **, $P<0.01$, for mannose between AKR/J mice and BALB/c control mice. The standard deviation of means are omitted for clarity. They were <10% of the mean.

patients with chronic lymphocytic leukaemia are shown in Table I. The results of these enzyme activities in the lymphocytes from 3 pools of peripheral blood from 5 donors for each pool are also shown in Table I as controls. MPI activity is lower than HK(M) in 4 of 6 patients studied with a HK(M)/MPI ratio higher than 1 (2.5, 2, 1.5 and 1.2 in each of the 4 patients indicated above). The ratios in the other two patients are similar to those in the controls (0.50±0.01). Similar activities are found in the patients and controls for HK(G) and GPI.

This marked shortage of MPI in 4 of 6 patients studied with chronic lymphocytic leukaemia, also found in splenic lymphoid cells from AKR/J leukaemic mice, could be a metabolic characteristic of several kinds of tumour cells, similar to that found in honeybees. If mannose is a selective toxic agent in those insects this sugar could also have the same effect in some tumour cells. So, given the harmlessness of mannose in healthy cells, this sugar seems to be a promising and supporting chemotherapic agent in patients whose tumour cells show a decreased MPI activity. However, further *in vitro* and *in vivo* studies on the effect of mannose in different tumour cells are necessary before suggesting the widespread use of mannose as a possible anti-tumour agent.

The authors thank Professor A. Sols for his valuable advice during the realization of the work. We also thank Dr A. Torres for peripheral blood from leukaemic patients, and Dr A. Galván for technical assistance.

### References

ARNOLD, H., SEITZ, V. & LOHR, G.W. (1974). Die hexokinase and die mannose-toxicität der biene. *Hoppe-Seyler's Z. Physiol. Chem.*, **355**, 266.

BEKESI, F.G., MOLNAR, Z. & WINZLER, R.J. (1969). Inhibitory effect of D-glucosamine and other sugar analogs on the viability and transplantability of ascites tumour cells. *Cancer Res.*, **29**, 353.

BERGMEYER, H.U., GAWEHN, K. & GRASSL, M. (1974). Enzymes as biochemical reagents. In *Methods of Enzymatic Analysis*, Bergmeyer, H.U. (ed) p. 425. 2nd ed. Academic Press: New York.

BOYUM, A. (1968). Isolation of mononuclear cells and granulocytes from human peripheral blood. *Scand. J. Clin. Lab. Invest.*, **21** (Suppt. 97), 77.

BUCHANAN, T., FREINKEL, N., LEWIS, N.J., METZGER, B.E. & AKAZAWA, S. (1985). Fuelmediated teratogenesis. Use of D-mannose to modify organogenesis in the rat embryo *in vivo*. *J. Clin. Invest.*, **75**, 1927.

DE LA FUENTE, M., PEÑAS, P.F. & SOLS, A. (1986). Mechanism of mannose toxicity. *Biochem. Biophys. Res. Commun.*, **140**, 51.

EAGLE, H., BARBAN, S., LEVY, M. & SCHULZE, H.C. (1958). The utilization of carbohydrates by human cell cultures. *J. Biol. Chem.*, **233**, 551.

FREINKEL, N., LEWIS, N.J., AKAZAWA, S., ROTH, S.I. & GORMAN, L. (1984). The honeybee syndrome. Implantations of the teratogenicity of mannose in rat-embryo culture. *N. Engl. J. Med.*, **310**, 223.

GROSS, L. (1951). Spontaneous leukaemia developing in C3H mice following inoculation in infancy with AK-leukemic extract or AK-embryos. *Proc. Soc. Exp. Biol. Med.*, **76**, 27.

HEREDIA, C.F., DE LA FUENTE, G. & SOLS, A. (1964). Metabolic studies with 2-deoxyhexoses. I. Mechanisms of inhibition of growth and fermentation in baker's yeast. *Biochem. Biophys. Acta.*, **86**, 216.

MORGAN, J.F. & ENG, C.P. (1970). Nutrition of mouse ascites tumor cells in primary culture. II. Specific requirement for glucose. *J. Natl Cancer Inst.*, **45**, 869.

SOLANA, R., SANTAMARIA, M., DE LA FUENTE, M. & PEÑA, J. (1982). Requirement of macrophage metabolic activity for T-lymphocyte activation. *Rev. Esp. Fisiol.*, **38**, 1.

SOLS, A., CADENAS, E. & ALVARADO, F. (1960). Enzymatic basis of mannose toxicity in honeybees. *Science*, **131**, 297.